# Supplementary material for: Identifying and understanding the nonlinear behavior of memristive devices
Source: Sci Rep. 2024 Dec 30;14:31633. doi: 10.1038/s41598-024-80568-y (PMC11686099; doi:10.1038/s41598-024-80568-y)
Supplement: Supplementary file 1 — Supplementary Information. [file 41598_2024_80568_MOESM1_ESM.pdf]

Supplementary material:

Identifying and understanding the nonlinear behavior of memristive devices

Sahitya Yarragolla[1,2], Torben Hemke[1], Fares Jalled[1], Tobias Gergs[2], Jan Trieschmann[2,3], Tolga Arul[4] and Thomas Mussenbrock[1]

[1]Chair of Applied Electrodynamics and Plasma Technology, Ruhr University Bochum, Universitätsstraße 150, 44780 Bochum, Germany
[2]Theoretical Electrical Engineering, Faculty of Engineering, Kiel University, Kaiserstraße 2, 24143 Kiel, Germany
[3]Kiel Nano, Surface and Interface Science KiNSIS, Kiel University, Christian-Albrechts-Platz 4, 24118 Kiel, Germany
[4]Chair of Reliable Distributed Systems, Faculty of Computer Science and Mathematics, University of Passau, D-94032 Passau, Germany

Flowchart of the proposed model:

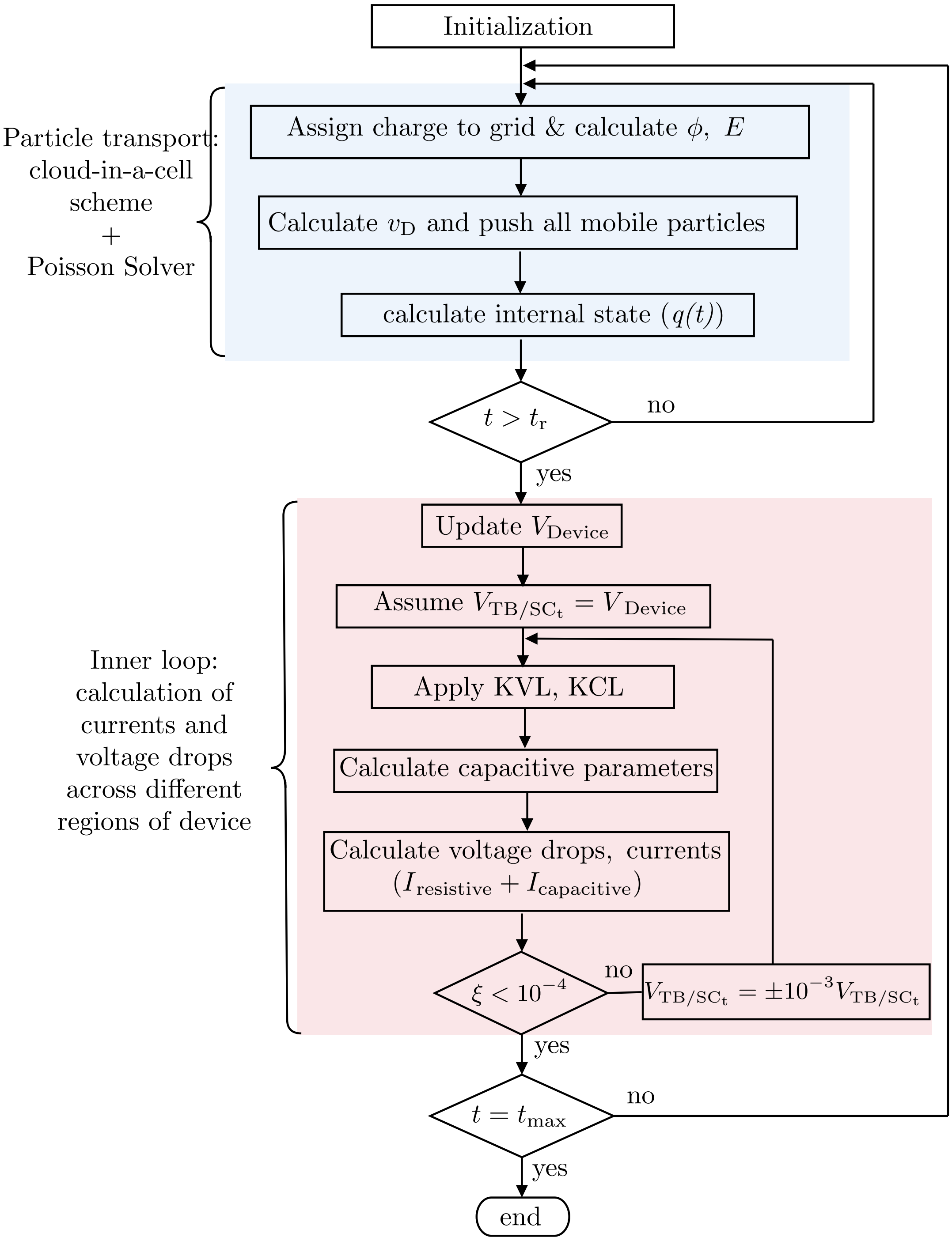

Figure S1: Flowchart describing all the steps involved in the proposed model

S II: Cloud-in-a-cell approach:

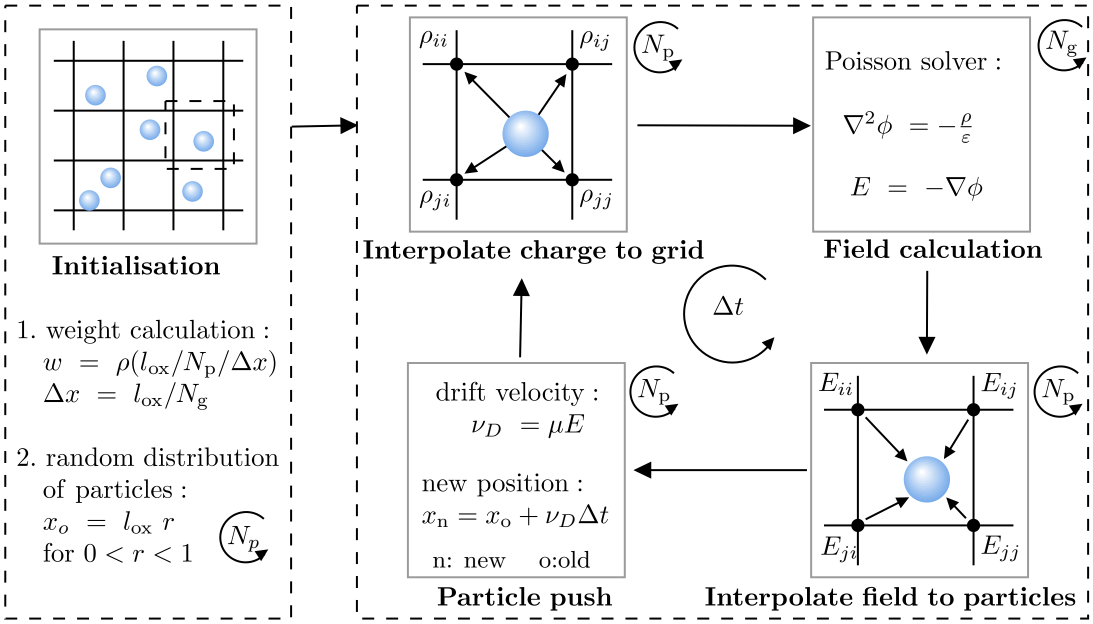

Figure S2: Flowchart describing the steps involved in cloud-in-a-cell (CIC) appraoch

The cloud-in-a-cell approach is a specific interpolation scheme that falls under the broader category of methods known as particle-in-cell (PIC) simulations. The CIC approach is used to study the behavior of particles (charged defects) in a metal oxide that contributes to resistive switching in RRAMs. In this approach, the RRAM device is discretized into a grid of cells (N_g) resembling a crystal lattice. Each cell represents a small volume within the device. The particles, representing atoms, ions, vacancies, or electrons, are conceptualized as forming a "cloud" around their actual positions. To distribute the properties of these particles onto the grid, the cloud around each particle is spread over multiple adjacent grid points. The initial position of each particle is given by:

$$x_o = l_{ox}\, r, \tag{S1}$$

where r is any random number between 0 and 1 and l_{ox} is the length of the oxide. The contributions of N_p particles are weighted based on their positions within the grid cells. In other words, particles are given weights based on the defect density (ρ) of the oxide. Here, weight refers to a value assigned to each simulated particle. The weight represents the number of real particles that the simulated particle represents. In CIC simulations, it is not practical to simulate every particle in an oxide layer, due to computational limitations. Instead, a small number of simulated particles are used to represent a large number of real particles. The weight of each simulated particle is determined based on the ratio of the total number of real particles in the oxide layer to the number of simulated particles. Mathematically, it can be represented by the below formula:

$$w = \rho \frac{l_{ox}\Delta x}{N_p}, \tag{S2}$$

with Δx as the distance between two grid points. Then, one critical aspect of the CIC approach is how the charge density at each grid point is calculated. The charge carried by particles influences the electric field within the device. To calculate the charge density, the particles' charges are distributed to the grid cells based on their positions. This process involves linear

interpolation techniques, where the charge from each particle is distributed among neighboring cells according to their position and weight. The charge density is calculated as follows:

$$\rho_{i/i+1} = \rho_{o,i/i+1} + eDw, \tag{S3}$$

where, D is the distance of the particles from their position to grid cells i or $i+1$, $\rho_{o,i/i+1}$ is the already present charge density (if any), and e is the elementary charge. This interpolated charge density is then used to update the electric field in the device. The simulation proceeds in discrete time steps. In each time step, the particles' positions and velocities are updated based on the forces they experience, such as electric fields. The complete process is further illustrated in Fig. S2.

In the study, we compared the computational performance of our cloud-in-a-cell scheme to a 0D model [1] and a 3D kinetic Monte Carlo-based model [2] for DBMD devices. The current-voltage characteristics and the computation time of different models developed for the DBMD device were analyzed (Fig. S3 (a)). The proposed 1D CIC model takes 20 s to run, compared to 2.8 s taken for the 0D model for the DBMD device and input voltage (Fig. 3f) . Despite the slightly longer time, the 1D model is more detailed, including stochasticity, spatially resolved potential and field, and ion transport. These features make it more accurate than the 0D model. While the simulation time of 20 s may be less suited for large-scale networks, it is ideal for detailed analyses of small circuits and single devices, balancing computational effort and physical accuracy. Additionally, while the kMC model takes around 9680 s, the 1D CIC model achieves comparable accuracy and efficiency in significantly less time. This efficiency of 1D CIC model is achieved through the use of superparticles for streamlined particle tracking, a one-dimensional grid for the Poisson solver to reduce complexity, and optimized Python libraries for enhanced solver performance. Fig. S3 (b-c) presents the profiler output of the DBMD device simulation using the 1D CIC model. This detailed breakdown highlights the computational time spent in various components of the simulation.

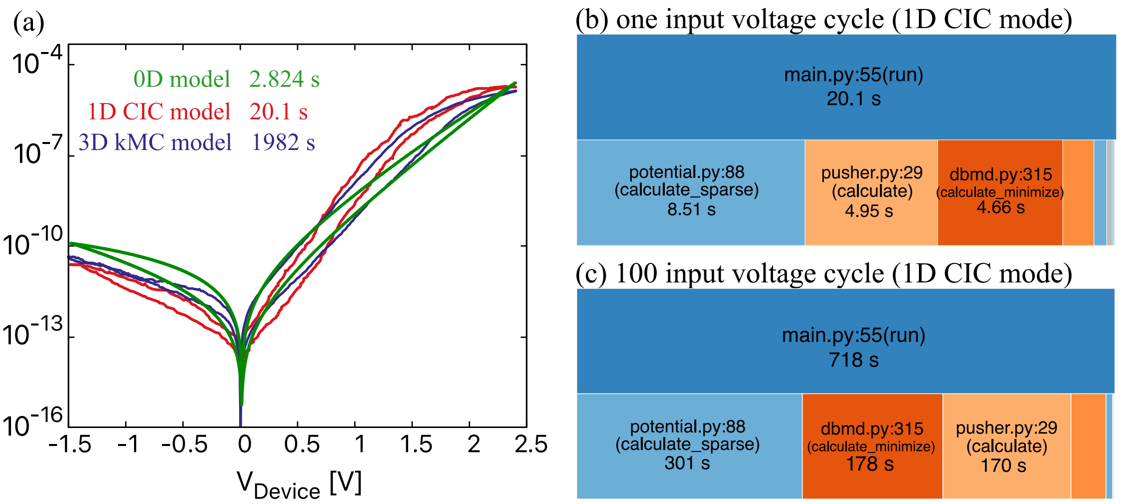

Figure S3: (a) The current-voltage characteristics and the computation time of different models developed for DBMD device. The profiler output of the computation of the DBMD device using the proposed 1D CIC model for (b) one and (c) 100 input voltage cycles. The main files are: main.py – the main file of the model, potential.py – the Poisson solver, dbmd.py – the calculation of currents, voltage drops, and capacitances, and pusher.py – ion transport. The ones that are not mentioned are the various other files that take comparatively very less time.

S III: Poisson Solver:

In many cases, particularly in this work, for the analysis of memristive devices, the 1D Poisson solver serves as a fundamental tool for calculating the distribution of electric potential and electric field in the oxide layer.

The 1D Poisson equation is derived from Gauss's law, which states that the divergence of the electric field in a region of space is proportional to the charge density within that region. Mathematically, it can be represented as

$$\frac{d}{dx}\left(\varepsilon\frac{d\phi}{dx}\right) = -\rho, \tag{S4}$$

where ε is the permittivity of the oxide layer and ρ is the charge density. The potential equation is subjected to Dirichlet boundary conditions at the simulation domain boundaries of the electrodes. For boundaries other than electrodes, periodic boundary conditions are applied. The Coulomb interactions between different particles are not taken into account. The electric field is then by

$$E = -\frac{d\phi}{dx}. \tag{S5}$$

To numerically compute the electric potential and electric field, Eq.(S4) and Eq.(S4) are discretized into a 1D mesh. The successive over-relaxation (SOR) method iteratively updates the solution to the Poisson equation. Eq.(S4) along the x-axis can be written as,

$$\phi_i^t = 0.5 * \frac{\rho\Delta x^2}{\varepsilon} + 0.5\left(\phi_{i+1}^t + \phi_{i-1}^t\right), \tag{S6}$$

$$\phi_i^t = (1-\omega_{\mathrm{SOR}})\phi_i^{t-1} + \omega_{\mathrm{SOR}}\phi_i^t. \tag{S7}$$

In this equation, the relaxation parameter,

$$\omega_{\mathrm{SOR}} = \frac{2}{1+\sqrt{1-cos^2\left(\frac{\pi}{N_{\mathrm{g}}}\right)}}. \tag{S8}$$

The electric field in the discretized form can be written as

$$E_i^t == \left(\frac{\phi_i^t - \phi_{i-1}^t}{2\Delta x}\right). \tag{S9}$$

S IV: Device parameters:

BFO device parameters used to obtain plots in Fig. 3(a)-(d), Fig. 4(a)-(b), Fig. 5 and Fig. 7(a).

**Physical quantity**	**Symbol**	**Value**
Temperature	T	298 K
Phonon frequency	ν_0	1×10^{12} Hz
Lattice constant	d	0.56×10^{-9} m
Device area	A_d	0.04 mm^2
Relative permittivity of $BiFeO_3$	ε_r	52.0
Activation energy de-trapping	U_A	0.55 eV
Activation energy trapping	U_A	0.76 eV
Conductivity of $BiFeO_3$	σ	7.0×10^{-4} Ωm
Length of BFO	l_BFO	600×10^{-9} m
Defect density	ρ	$8 \times 10^{16}\ \mathrm{cm}^{-3}$
Top Schottky contact height	$\Phi_{\mathrm{SC_t}}$	0.75 eV
Top Schottky contact ideality factor	$n_{\mathrm{SC_t}}$	4.0
Fitting parameter top SC depletion layer	$\lambda_{\mathrm{d_t}}$	0.3
Bottom Schottky contact height	$\Phi_{\mathrm{SC_b}}$	0.85 eV
Bottom Schottky contact ideality factor	$n_{\mathrm{SC_b}}$	4.42
Fitting parameter bottom SC depletion layer	$\lambda_{\mathrm{d_b}}$	-0.9

Table S1: Details of the simulation parameters of BFO device.

DBMD device parameters used to obtain plots in Fig. 3(e)-(h), Fig. 4(c)-(d), Fig. 6 and Fig. 7(b).

**Physical quantity**	**Symbol**	**Value**
Temperature	T	298 K
Lattice constant	d	2.5×10^{-10} m
Device area	A_d	625 $\mu\mathrm{m}^2$
Relative permittivity (Nb_xO_y)	ε_r	42.0
Activation energy	U_A	0.76 eV
Conductivity (Nb_xO_y)	σ	1.0×10^{-4} Ωm
Length of SE (Nb_xO_y)	l_SE	2.5×10^{-9} m
Defect density	ρ	$5 \times 10^{20}\ \mathrm{cm}^{-3}$
Tunnel barrier width	d_TB	1.1×10^{-9} m
Tunnel barrier height	Φ_TB	3.2 eV
Fitting parameter TB depletion layer	λ_t	0.1
Schottky contact height	Φ_SC	0.96 eV
Schottky contact ideality factor	n_SC	4.2
Fitting parameter SC depletion layer	λ_d	-0.7

Table S2: Details of the simulation parameters of DBMD device.

S V: Frequency spectra of BFO similar to Fig. 5, different voltage amplitudes

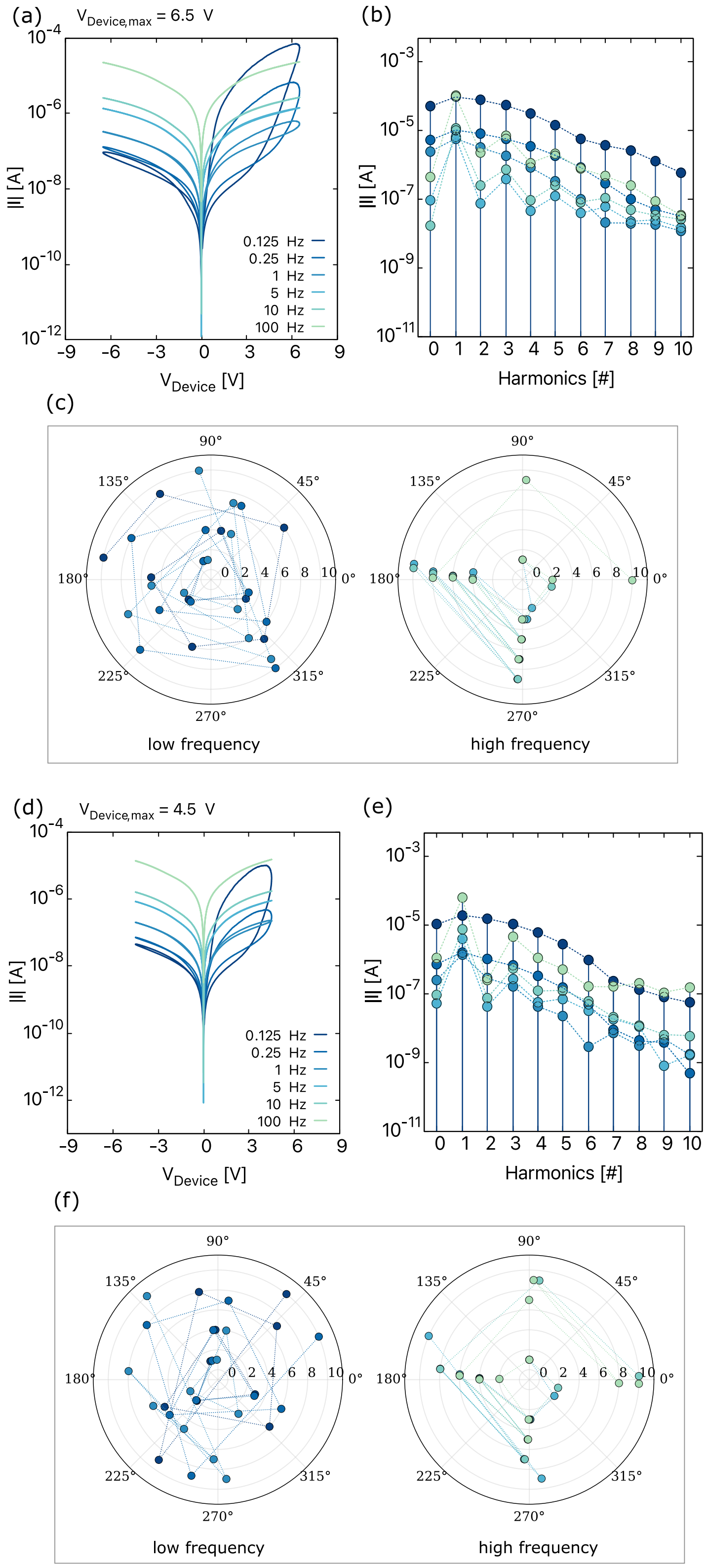

Figure S4: (a),(d) The simulated current-voltage characteristics of BFO obtained for a sinusoidal input voltage of 6.5 V and 4.5 V with various frequencies, Frequency spectra of BFO corresponding to the I-V curves depicted in Figure (a) and (d) showing (b),(e) amplitude spectra and (c),(f) phase spectra.

S VI: Frequency spectra of DBMD similar to Fig. 6, different voltage amplitudes

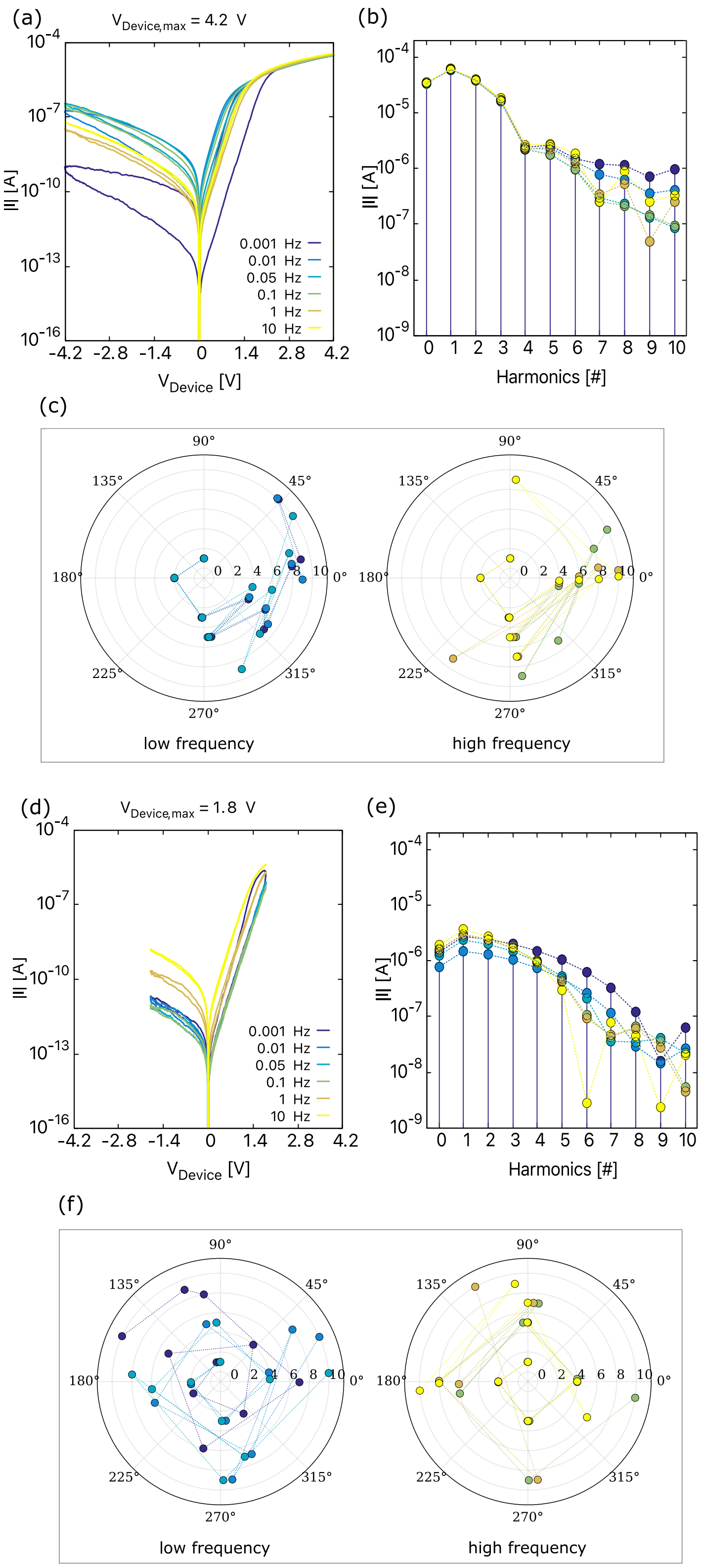

Figure S5: (a),(d) The simulated current-voltage characteristics of DBMD obtained for a sinusoidal input voltage of 4.2 V and 1.8 V with various frequencies, Frequency spectra of DBMD corresponding to the I-V curves depicted in Figure (a) and (d) showing (b),(e) amplitude spectra and (c),(f) phase spectra.

References

[1] Hansen, M., Ziegler, M., Kolberg, L. et al. A double barrier memristive device. Sci Rep 5, 13753 (2015).

[2] Dirkmann, S., Hansen, M., Ziegler, M. et al. The role of ion transport phenomena in memristive double barrier devices. Sci Rep 6, 35686 (2016).
